# Adolescents’ reproductive health knowledge, choices and factors affecting reproductive health choices: a qualitative study in the West Gonja District in Northern region, Ghana

**DOI:** 10.1186/s12914-018-0147-5

**Published:** 2018-01-24

**Authors:** Joseph Maaminu Kyilleh, Philip Teg-Nefaah Tabong, Benson Boinkum Konlaan

**Affiliations:** 1 Nurses Training College, Box 565, Tamale, Northern region Ghana; 2 Department of Social and Behavioural Science, School of Public Health, Box LG 13, Legon, Ghana; 3 Department of Community Health and Family Medicine, University for Development Studies, Tamale, Ghana

**Keywords:** Adolescents, Reproductive health, Health choices, Risky behaviours, Abortion, Ghana

## Abstract

**Background:**

In Ghana, adolescents constitute about a quarter of the total population. These adolescents make reproductive health decisions and choices based on their knowledge and the availability of such choices. These reproductive health decisions and choices can either negatively or positively affect their lives. This study therefore explored adolescents’ reproductive health knowledge and choices, the type of choices they make and the factors that affect these choices.

**Methods:**

This qualitative study adopted a narrative approach to qualitative enquiry. Eight focus group discussions (*N* = 80) were conducted among both in-school and out-of-school adolescents aged 10–19 years. The discussions were stratified by sex and studentship. In addition, nine in-depth interviews were conducted with various stakeholders in reproductive health services and community opinion leaders. Both the focus group discussions and in-depth interviews were recorded, transcribed and analysed using NVivo 11. Thematic analysis was employed in analysing data.

**Results:**

The study found that knowledge on reproductive health choices was low among respondents with majority of them relying on their peers for information on sexual and reproductive health. Having a sexual partner(s) and engaging in premarital sex was common and viewed as normal. Adolescents engaged in unprotected sexual practices as a way of testing their fertility, assurance of love, bait for marriage and for livelihood. Inserting herbs into the vagina, drinking concoctions and boiled pawpaw leaves were identified as local methods employed by adolescents to induce abortion. Reproductive health services were available in the community but received low utilization because of perceived negative attitude of health workers, confidentiality and social norms.

**Conclusions:**

Adolescents in this study generally engaged in risky reproductive health choices that can negatively affect their reproductive health. Adolescents in this part of Ghana have challenges utilizing available reproductive health services because of socio-cultural and health system barriers.

## Background

Adolescence is a period of life during which individuals reach sexual maturity [1]. It is the period of transition from childhood to adulthood and it is often characterized by biological and psychosocial changes as well as sexual experimentation [2]. Globally, adolescents constitute about one billion of the world’s population, with 70% living in developing nations [3]. In sub-Saharan Africa, young people constitute about 33% of the 973.4 million population. The population of adolescents and young adults is expected to continue to increase over the next 35 years [4, 5]. In sub-Saharan Africa, where a fourth of all adolescents are reported to have sexual experience, education on sexual and reproductive health are generally reported to be low [3]. In Ghana, the 2010 Population and Housing Census reported that the ratio of adolescents between 10 and 19 years to the total population is 1: 4.5; meaning this age group constitute about 22.4% of the national population [6]. In the northern region of Ghana, adolescents between 10 and 19 years are about 22.3% of the regional population and about 10.1% of the national adolescents’ population [7].

Evidence abound that adolescents experience very critical and life defining events, namely; first marriage, first sexual intercourse, and parenthood [8]. The downward trend in age at menarche from 15.5 years [9] to an average of 12–13 years in most developing countries [10] also means an increase in the interval between menarche and marriage. According to the Ghana National Population Council, the age at first marriage was 18.3 years for females and around 25 years for males in 1988, but this age has increased to around 21.4 years for females living in urban areas and 20.9 years for their counterparts in rural areas. For males, it increased to 26.1 years (urban residents) and 24.9 years for rural dwellers [11].

Adolescents’ knowledge and access to reproductive health services is important for their physical and psychosocial wellbeing. It has been found in an earlier study that the lack of knowledge about the consequences of unprotected premarital sex among adolescent females predisposed them to unwanted pregnancies, unsafe abortion and its complications, and sexually transmitted infections [12]. According to the 2014 Ghana Demographic and Health Survey (GDHS), about 14% of females aged 15–19 years had begun child bearing. Of these 14%; about 11% have had a life births and 3% were pregnant at the time of the survey [13]. Abstinence, use of condom, use of contraceptives, decision to keep a pregnancy, use of safe abortions services are some of the choices and reproductive health decisions adolescents make [14]. The International Conference on Population and Development (ICPD) which was held in Cairo in 1993 recognised the negative effects of risky sexual behaviour. Several countries including Ghana were implored to institute measures to ameliorate the situation [15]. Therefore, adolescent-friendly reproductive health services and comprehensive abortion care were instituted in Ghana to increase access to reproductive health and safe abortion services. This notwithstanding, many adolescents still encounter significant obstacles in accessing sexual and reproductive health services [16]. Knowledge on reproductive health services is essential to enable them make informed choices. The type of choices made by these young adults could either impact positively or negatively on their lives, their families and the society at large [15]. This study therefore explored adolescents’ knowledge about reproductive health and choices, what choices they make and the factors that affect those choices.

## Methods

### Study design

**Fig. 1:**
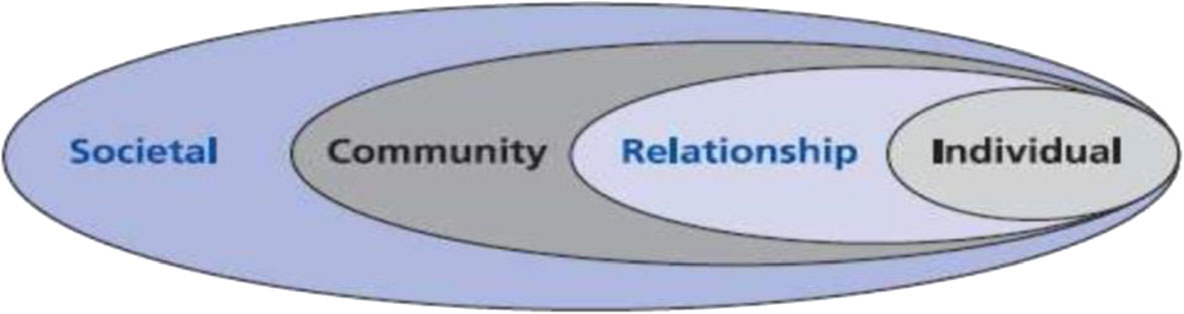
Ecological model for adolescents’ reproductive health choices and factors influencing the choices [20]

This study adopted a narrative approach to qualitative enquiry. Narrative research allows participants in a study to share their experiences in the community [17]. Since the researchers were interested in exploring the adolescents’ knowledge on reproductive health services and choices, factors affecting their reproductive health choices, and how these choices affects their lives, the narrative approach was deemed appropriate [18]. We conducted focus groups discussions with adolescents and individual interviews with stakeholders. In this study, we adopted the ecological model (Fig. 1). The ecological model provides a framework for understanding the multiple and interacting factors of adolescent sexual and reproductive health behaviour and their effects [19, 20]. This framework posits that adolescent sexual and reproductive health behaviour and their choices are influenced by interpersonal, organisational, community and public policy factors. This model recognises that these factors (interpersonal, organisational, community and public policy) interact across different levels, focus on specific health behaviour and that interventions that address the multiple levels are more effective [21]. In the entire research process, steps were taken to adhere to the requirements of RATS guideline for conducting and reviewing a qualitative research [22].

### Study area

The study was conducted in the West Gonja district of the Northern region of Ghana. The West Gonja district is one of the 26 districts in the region which lies within the savannah belt. The district has an estimated population of 46,803 with an annual growth rate of 2.9%. There are four major religious groups in the district, namely: Islam (about 70%), Catholics (10%), Protestants (8%) and Traditional Worshippers (12%) [23]. The 2011 and 2012 annual reports of the West Gonja District Health Directorate showed that the district recorded 13.7% and 14.4% of teenage pregnancies with 9.9% and 11.4% delivery rates respectively.

### Selection of participants and data collection

The participants in this study were adolescents aged 10–19 years who lived in the West Gonja District at the time of the study. Both male and female in-school and out-of-school adolescents were recruited for the study. The communities were selected based on two criteria; high school dropout rates and teenage pregnancies. The researcher first collected the annual reports from the district and based on that communities were grouped into two; those with high teenage pregnancy and school dropout rates; and those with low teenage pregnancies and school dropout rates. From each category four communities were selected where the study was conducted. At the community level, school authorities were contacted for approval to recruit in-school adolescents. However, for the out-of-school participants, these were selected through the assistance of community leaders.

For stakeholders in adolescents’ sexual and reproductive health, a purposive sampling technique was used. In purposive sampling technique, researchers choose the sample based on who they think are appropriate for the study [24]. Hence, community opinion leaders and health workers who provide adolescent reproductive health services were selected.

Two main data collection strategies were employed in this study namely; focus group discussions (FGDs) and in-depth interviews (IDIs). Focus group discussion (FGD) refers to a qualitative data collection method where between 6 and 10 individuals with similar background or experiences are brought together to discuss a specific topic of interest with a researcher [25]. Thus, the use of focus group allows small number of participants to discuss a study topic led by a moderator using a discussion guide [26]. The focus discussion groups were homogenous for sex and studentship. The FGDs aimed at capturing the local context of sexual and reproductive health of the adolescents and, to enable the investigators get a true picture of the social reality. The FGD guide focused on individual, relationship, and community level factors that affect adolescents’ reproductive health knowledge and choices as required by the ecological framework used for this study. With the aid of an interview guide the investigators introduced the topic to the group and gave them leeway to express themselves. Their responses gave room for further probes. Participants’ responses were written in a field note book and recorded using a digital recorder as well.

Eight FGDs were conducted; four among in-school adolescents (2 males, 2 females) and four among adolescents who were out of school (2 males, 2 females). Each group comprised of 10 discussants making a total of 80 participants in all. Some of the adolescents were married whilst others were not married.

In-depth interviews using semi-structured topic guide were conducted with individuals who were engaged in reproductive health services in the district as well as opinion leaders in the community. This was done to elicit information on both community and the health service related factors that may inform the choices that adolescents make. In all nine IDIs were conducted among various stakeholders. The stakeholders were: one medical doctor, one public health nurse, two midwives, two community health nurses, two community opinion leaders (1 male, 1 female) and the programme manager of World Vision International, a non-governmental organisation engaged in child and adolescent educational programmes in the study area. The data collection was ended at the point of saturation as required in qualitative research [24]. The IDI topic guide covered areas such community and health system related factors, policies and strategies to ensure safe reproductive health practices among adolescents in line with the societal construct in the ecological model.

### Data analysis

Data collected during the FGDs and IDIs were digitally recorded and transcribed verbatim. The field notes were converted into data documents. All transcripts were reviewed by an independent person who is an experienced qualitative researcher. In the review, the independent person listened to the recorded voices and compared the voices with the transcripts. Qualitative narrative data in English were then entered into a word processor (Microsoft Word) and imported into NVivo 11 for analysis.

Thematic analysis was employed in analysing the data. Thematic data analysis process involves data reduction, data display and data conclusion-drawing/verifying [27]. Line-by-line coding of the various transcripts were done as either free nodes or tree nodes. Queries (analysis in Nvivo) were performed to compare the coding against nodes and attributes to compare and contrast within-group and between-group responses and themes.

## Results

### Knowledge on reproductive health services and choices

The results from the study revealed that both in-school and out-of-school adolescents had little or no understanding of adolescent reproductive health services and choices. However, some adolescents (15 in-school, and 6 out-of-school) were able to identify abstinence, use of condoms, and other contraceptives to prevent unplanned pregnancies. The following quotes illustrate the understanding of participants as to what reproductive health services are all about:

*“Reproductive health services are the service that teach us how to protect ourselves from getting pregnant through the use of condom during sex…also use contraceptives though it is sometimes difficult for us to use contraceptive”* (female, FGD, in-school).

*“As adolescents, we have to make choices between not having sex until we are married, or if we cannot wait we have to use contraceptive methods that would prevent us from becoming pregnant”* (Female, FGD, in-school).

### Sources of reproductive health knowledge

FGDs respondents identified peers, parents, teachers, radio and television as the main sources of information on reproductive health. However, most of the adolescents especially those out of school relied mostly on their peers for information on reproductive health. The results showed that parents were an important source of information. For in-school adolescents, teachers emerged as another important source of information on reproductive health as it was unanimous among FGD participants. The following are quotes from some participants to illustrate these views:

*“….the major sources of information are through our teachers and nurses. Most of us actually prefer getting our “filla” [information] from our friends and sometimes nurses”* (female, FGD, in-school).

*“…we get some information from the schools we attend. Sometimes too our parents give us some of the information we need in the form of a warning. I think most of the time when we discuss about it with friends we get to know more about the issues”* (male, FGD, in-school).

*“When we meet our friends who are more experienced they teach us how to protect ourselves from becoming pregnant. If you have a problem then you bring it out and people will advise you appropriately”* (female, FGD, out-of-school).

The study also found that health workers generally believed that there was the need to provide adolescents with reproductive health information. Therefore health education sessions are organized in schools by trained nurses to talk to the students about sexual and reproductive health matters. This opinion is captured in the following statements:

*“We have arranged with the schools so that from time to time we give them health talks* on *sexual and reproductive health”* (Midwife-1, IDI).

*“In one of the communities here, we noticed that teenage pregnancy was very common resulting in high school dropout rate among female adolescents. So we organised to go and educate them and also provide them with some contraceptives at the school, but the school authorities did not agree. When the community heard about it, they sent a delegation to warn us to desist from such acts. They said we wanted to encourage premarital sex. But you see, the teenagers were becoming pregnant and when you ask about the one responsible, you see it is usually an adult not a colleague teenager”* (Public Health Nurse, IDI).

### Views on having sexual partners and premarital sex

The findings of this study showed that having multiple sexual partners was considered a source of pride among both male and female participants. It emerged also that while the adolescent male had adolescent female as partners; majority of their counterparts (females) had adult males as partners. The following are quotes from the FGDs to buttress these points:

*“As for boyfriends most of us have them, and some girls have sexual intercourse with their boyfriends. So teen pregnancies among the youth are uncountable around here” (*female, FGD, in-school).

*“Abstinence among the youth is very difficult. Some people try to abstain from having intercourse, but they are usually described in derogatory terms such as; your penis is not good or manhood is not working” (*male, FGD, in-school).

*“For us males, our sexual partners are our colleagues but for the females, their partners are mostly adults. So it is common to find a female having multiple partners because they will have one schoolmate as a boyfriend and an adult who will be providing her with material things and money” (*male, FGD, out-of-school).

Furthermore, both adolescents and stakeholders in this study perceive that sexual activities was rife among both in-school and out-of-school adolescents. The reasons adduced for adolescent engaging in sexual activities include: for sexual pleasure, to comply with his/her group norms, for gifts and also as an expression of love to their partners. Though adolescents in this study acknowledged that premarital sex is risky, it was equally generally believed to be worth the risk and therefore perceived to be indispensable. It also emerged that having sex with multiple partners by female adolescents was common especially sexual activities for gifts or favour from men. The following quotes support these points:

*“Their expectations are that they will marry each other. Some expects to have fun and feel good as a girl or boy. Others expect support like money, gifts, clothing and other things from the boyfriend or the girlfriend. Some too expect trust from their girlfriends such that the girl should not have any other boy as a friend”* (male, FGD, out-of-school).

*“Yes the risk is there because one can get pregnant by having sex but we still do it. You can also get other sicknesses by having sex but what can we do? We have to do it to get what we want”* (female, FGD, in-school).

*“For us girls, it mostly for gifts from adults. Sometimes, there is nothing you can do about it because that adult is the one taking care of you so you risk losing him to another girl if you do not oblige. My first sex was with a man taking care of me. When I wanted to resist, he threatened to stop taking care of me and get another girl. So because of that I agreed”* (female, FGD, in-school).

Interview with community opinion leaders revealed that this practice was really common and many female adolescents relied on it for their upkeep and also to take care of their education. Despite acknowledging this fact, opinion leaders we interviewed believed that advocating for the use of contraceptives was not the way to go. In their view, modern contraceptives can cause infertility among female users. To community opinion leaders, men who are not biological parents to adolescent females take care of their educational needs with the intention of marrying them in future. Also, a woman’s ability to beget children for the husband was perceived as a reward for the investment the man made in her education. So, with the belief that modern contraceptives could cause infertility, their use was seen as something that could lead to a loss in the man’s investment in the adolescent female. The following quotes illustrate these points:

*“Premarital sex is very common in the community and for the girls, it is the adults that take them as their partner. Because of poverty, the girls have to rely on these adults for money and upkeep, so they take advantage of them”* (Female, Opinion Leader, IDI).

*“As for the contraceptives, the men will not agree because, it is believed that it can cause infertility in future. Men in this community take care of female adolescents to marry and have children with them in future. So, if the girl uses contraceptives and become infertile in future, it will mean the man has invested in vain. It is a serious problem, so some NGO is assisting the girls”* (Male Opinion Leader, IDI).

The study also explored the use of condoms during such premarital sex since the use of female contraceptives were deemed inappropriate. Adolescents in this study believed most of them engage in unprotected sex. Condoms were believed to inhibit the pleasure in sex and since many engaged in sex for pleasure, the use of a condom was also regarded as impracticable. Another reason for the non-use of condom during sex was the inconvenience or challenges involved in getting one. Some adolescents were of the view that it was difficult going to buy condoms. This is because an adolescent who goes to buy a condom will be perceived as a “bad boy or girl” In their opinion, many of the drugstore sellers in the community know their parents. Therefore they were afraid the sellers may convey that information to their parents. The following quotes illustrate these points:

*“Oh yes, condom is a waste of time and no feelings. Everything in life there is a risk and sex itself is a risk. The risk is there in having sex because most do not use condoms; some too have about two or more girlfriends and always have sex with all of them. Through that you can get any disease or even impregnate a girl that you may not even like to marry or have a child with”* (male, FGD, out-of-school).

*“Yes sometimes when you want to have sex you tell the boy to use condom. Some males agree and use but there are some males who will tell you that if you put a toffee with the wrapper in your mouth do you get the sweetness of the toffee?” (*female, FGD, out-of-school).

*“You know, the condoms are sold at drug stores so when you want it, it is difficult to go there and buy especially us the girls. Yes, because of your age some say, you are too small to buy condoms”* (female adolescent, FGD in-school).

*“….Young people feel shy or afraid to buy condoms because the chemist shop owner may go and tell your parents that you have started using condoms or having sex”* (male adolescent, FGD in-school).

### Common strategies adopted to prevent pregnancy

This study explored what adolescents do to prevent getting pregnant. The results revealed that local remedies were available and widely used by community members. One of the strategies adopted by adolescents to prevent pregnancy is the use of a local herb called *“yigewulso”.* This herb is believed to have contraceptive effects. Other herbs also believed to have similar effects are used as emergency contraceptives after unprotected sex. This study also found that some adolescents believed that wearing of some local beads around the waist during sexual intercourse could prevent a pregnancy outcome. The following quotes serve to illustrate their views:

*“We have this herb called “yigewulso” which is usually taken before sexual intercourse if you don’t want to get pregnant”* (male, FGD, in-school).

*“If you have sexual intercourse and you don’t want to be pregnant, you have to take “kaligutim” immediately. Normally, we buy it from the local chemist shop. It can be used either as an emergency contraceptive or when you miss your period”* (Female, FGD, Out-school).

*“In this community there is a belief that you can prevent getting yourself pregnant if you wear beads in your waist during sexual intercourse. This is why most girls wear beads around the waist before they have sex. Even women who are breastfeeding babies also wear beads to avoid pregnancy while the child is still young”.* (IDI, Midwife-1).

These adolescents also reported that other techniques they employed to prevent pregnancy outcome was for the female to lie in the prone position or wash her vagina with soap and water immediately after sexual intercourse. These practices in their opinion would evacuate or kill the sperms in their vagina. They were also of the view that these practices were safe and produced no adverse effects. They had this to say:

*“I was told by my friend that when you wash your vagina with soap and water and also lie on your stomach (prone position) immediately after sex, you won’t get pregnant. So, we do it to prevent pregnancy”* (female, FGD, out-of-school).

“*..Some of the traditional methods are better. If your girlfriend knows them and practice them there is no way she will get pregnant, and won’t have problems like those who use the modern contraceptive method”* (male, FGD, out-of-school).

### Unplanned pregnancies, abortions and sexually transmitted infections

Participants in this study were of the view that unplanned pregnancies were common among adolescents in the district. It emerged that some adolescents in this community believed that getting oneself pregnant was the guarantee or proof of one’s fertility. Male adolescents will also test their manhood by insisting on having unprotected sexual intercourse with the partner and hoping to be told she had “missed her period” (meaning she is pregnant). Sometimes the females may also prefer to have unprotected sexual intercourse in the hope of becoming pregnant as a bait for marriage as well as test for future fertility. The following quotes support these assertions:

*“A lady became pregnant and she decides to abort it because she has nobody to take care of the baby. Another lady became pregnant for a guy she loved but her parents disapproved of their relationship because the boyfriend was not doing any work”* (Female, FGD, out-of-school).

*“Their knowledge level is little. This is because, data gathered in the district indicates high rate of teenage pregnancy and sexually transmitted infections” (*IDI, Midwife-2).

*“In this community some of the girls try to get pregnant intentionally to show that they are fertile. Even a girl can tell the colleague you have been having sex with your boyfriend without ever becoming pregnant, it means either you or boyfriend is infertile”* (IDI, Opinion Leader).

The results from this study also suggest that most of these unplanned pregnancies are aborted through unsafe practices using a combination of methods such as drinking concoctions of boiled pawpaw leaves, Nescafe, grinded bottles, alcoholic beverages and inserting herbs into the vagina. Participants in this study were of the view that these methods of terminating pregnancy are widely used in the community. The information gathered by this study suggest that some of the unsafe abortions have often resulted in fatal outcomes as illustrated by the following quotes:

*“In fact we have plenty illegal abortion in this district particularly in Damongo town. For example one girl just died here last week. What we found out later was that she was given grinded bottles to drink. It is a very common practice”* (IDI, Midwife).

*“….A friend recently got pregnant and decided to abort using “Salaamalekum” leaves [herb] to do the abortion. Some also use some type of fruits, pawpaw leaves, Nescafe and sugar, alcoholic and non-alcoholic drinks as well as broken bottles to cause the abortion” (*female, FGD, out-of-school).

*“I know a girl who was pregnant and the boyfriend bought malt and mixed it with grinded bottle and gave it to her and she drank, few hours after drinking that she bled and finally died”* (female, FGD, in-school).

Participants in this study also indicated that sexually transmitted infections (STIs) were common in the community. They attributed this to the youth practicing unprotected sexual intercourse with multiple partners. In their opinion, there is risk in every activity. So it was normal to enjoy sexual intercourse and treat any STI that may arise. The following quotes illustrate these points:

*“Sexually transmitted infections especially white (candidiasis) is very common among the females in the area. Often when they come they will just say…madam I have white and we have to test them for STIs”* (IDI, Midwife-3).

*“The STIs you are talking about is very common in this community because many of the youth have multiple partners which they call it “inter” and “exter” one in your school and one outside your school”* (male, FGD, in-school).

*“There is risk in everything we do, so it is better to enjoy yourself during sexual intercourse and if you get a disease, you treat it. I don’t ask the man to use condom, I don’t like it myself”* (female, FGD, in-school).

### Access to reproductive and sexual health services in the community

The findings of our study show that reproductive health services are generally available in the community. There are outreach services for the communities. These include; health education, counselling, and provision of contraceptive services. The study also found that adolescents were generally aware of some of these services and how to access them except for comprehensive abortion care. The following quotes illustrate these points:

*“We have service-points for adolescent reproductive health service in this district where we provide education, counselling, contraceptive service and comprehensive abortion care to adolescent who visit us”* (IDI, Public Health Nurse).

*“There are outreach services for communities where there is no clinic or hospital to provide reproductive health service”* (IDI, Midwife-2).

*“The nurses have been coming to tell us that we can come to them for reproductive health service, so we know those services are available at the hospitals and clinics”* (female, FGD, in-school).

The above notwithstanding, the results of this study show that there were some barriers to accessing these services. Four main sub-themes emerged in this respect, namely; sociocultural, attitude of service-providers, lack of privacy and confidentiality on the part of service providers and finally perceived adverse effects. The study found that the community generally perceived issues about sexual and reproductive health as only suitable for adults. Therefore, it was the general believe that it would be inappropriate to discuss such issues with adolescents. Though health workers were trained to provide these services to adolescents, there was the general believe and fear among adolescents that they may be scolded or described as “bad boys or girls” if they should go for such services. Some adolescents were also of the view that health care providers do not treat adolescents who seek for reproductive sexual health services well. The poor attitude of health providers towards adolescents deters the later from patronizing the available services.

Another barrier reported in this study was the lack of privacy. Adolescents who participated in this study were of the view that the service points did not provide enough privacy and confidentiality. In their opinion, the present environment does make it possible for people to see adolescents who seek such services as ‘spoilt children’ or may even conclude that you have come to do abortion or contraception. Another issue of confidentiality was the general believe that service providers sometimes tell parents of adolescents who seek such services that their ward was sexually active. The following quotes support these points:

*“In this community, if an adolescent ask about sexual and reproductive issues, the person will be seen as a bad girl or boy. So we are afraid to go to the clinic and hospital for such services. Imagine a girl going to the hospital to do something and the information comes out that you went there to do abortion. People will say you are bad, and if you are not lucky, the information could spread to all over town the next day”* (female, FGD, out-of-school).

*“… Some of the nurses are not polite especially to us the young people. You go there with a problem then they will be shouting at you or even insulting you saying you are bad girl or boy. The authorities should punish or even sack such people, but you see, some of us do complain about these things but nothing happens to them”* (Male, FGD, In-school).

Interviews with services providers revealed that the design of some of the service points makes it difficult to provide optimum privacy to clients. The study also found that some staff were not trained on adolescent friendly reproductive health services as illustrated:

*“…The lack of privacy is due to how our facilities were designed. Many of our facilities in this district have no space provided for that. Lack of confidentiality from health personnel, as well as inadequate qualified personnel are serious challenges that we face on the daily basis”* (IDI, Midwife-1).

*“Our staff are not well trained as adolescent friendly reproductive health service providers. We are all using our previous knowledge from school which may not be enough and also the working experiences… everybody is committed in helping out to make the situation better. Inadequate staff numbers is also a big problem for us. The work is, actually too much for us”* (IDI, Midwife-2).

*“….Our consulting rooms are not safe. When a person comes with a sexually transmitted infection, they often not able tell us because some people may hear the conversation. They rather try to hide the truth from you and begin to tell you about other things, leaving you to guess the problem”* (IDI, Public Health Nurse).

Stakeholders interviewed in this study noted that access to SRH information and services could reduce school dropout rate among females. However, stakeholders indicated that some health workers were uncomfortable providing adolescents with reproductive health services. In their view, availing condoms to adolescents may lead them to experiment with sex. Also, increasing access to comprehensive abortion services will encourage sexual activity among adolescents. Some stakeholders were of the view that some health workers have a negative attitude towards comprehensive abortion service because it conflicts with their religious beliefs. The following quotes illustrate these points:

*“Allowing adolescent access to condom will encourage sexual practice among them. As for access to abortion, it means we will be encourage them to experiment with sex. After all when you become pregnant you can abort it at the hospital”* (IDI, Opinion Leader).

*“Some health workers are uncomfortable providing comprehensive abortion care to adolescents. They perceive abortion as something that is against their religious beliefs. So, it is even worse when they are to provide such a service to adolescent”* (IDI, Public Health Nurse).

## Discussion

### Knowledge on reproductive health and sources of information

The study found that both in-school and out-of-school adolescents in this part of Ghana did not have comprehensive knowledge on reproductive health issues and choices. The lack of knowledge makes them vulnerable to unsafe reproductive health behaviour and inappropriate choices. Some of these choices may have detrimental effects on their reproductive health and future. For example, a wrong choice can lead to unplanned pregnancy or STI infection [28]. In another study, it was found that lack of knowledge on reproductive health was associated with early initiation of coital relations and of unwanted pregnancies [29]. The effects of these unplanned pregnancies are multifarious with some capable of lasting for a lifetime. These potential human resource and future leaders end up as school dropouts due to unplanned pregnancy and other attendant complications. Additionally, a good number of adolescents who indulge in early sexual debut may contract HIV and other STIs [30]. These have social and economic implications for their households and the nation as whole as funds will be required to provide lifetime medication for people with HIV [31], and may even affect their line of generations yet unborn [32].

As we have shown, most out-of-school adolescents are reliant on their peers who are in-school and the mass media for information on reproductive health. These sources make them vulnerable to misinformation. In that case, they will be making decisions based on an incorrect information which can negatively affect them. Parents who could be the most appropriate source of information are inhibited by socio-cultural barriers that prevent them from discussing reproductive health issues with their children as has been reported by Owusu, Blankson & Abane [33] in the Central Region of Ghana. Similarly, studies in Nigeria and Uganda found that adolescents preferred parents as a source of information about sexual and reproductive health, however cultural sensitivity and social norms inhibited them [parents] from playing that role effectively [34–36]. Talking about sex is often frowned upon by both traditional and religious adherents in the Ghanaian society. This environment makes it difficult and sometimes impossible for adolescents to discuss sex and related issues with parents or adult family members [37]. The findings of this study underscore the need for innovative ways to expand access to reproductive health education and services to both in-school and out-of-school adolescents. School-based approaches which are linked to the community have been found to be effective in other countries [21, 38, 39]. These approaches could be adopted for Ghanaian adolescents as a community-related strategy (intervention) in the ecological model.

### Views on having sexual partners and premarital sexual practices

The study found that having a sexual partner was a common practice among adolescents in the community, and is widely viewed as an acceptable practice. Among adolescents, this is done to conform to peer norms and a way of demonstrating that one had what it takes to be a woman or man. The act of engaging in sexual practice among adolescents has been widely reported across the sub-Saharan African region, with about 25% reporting having sexual contact before attaining 15 years of age [40].

This study also found that having multiple partners was a common practice. Similar findings were reported among adolescents in Tanzania [41]. Despite the fact that many adolescents reported having multiple partners, the use of condom was reportedly low during sexual encounters. This is a challenge to public health workers involved in sensitizing the population against risk of STIs and HIV among Ghanaians. The use of condom is one of the key strategies employed by the National AIDS Control Programme (NACP) to reduce the burden of HIV and STIs. Having sex with multiple partners without the use condom is one of the risk factors in HIV transmission and many studies have documented high prevalence of HIV infection among people with multiple partners [42–44].

The study found that transactional sex (sex for gift) was common in the community and many adolescents were engaging in this type of sexual acts with adults in the community. Female adolescents were engaged is this practice as a way of survival as a result of endemic poverty in the community [45]. This will require interventions at the community level to empower females. Also enforcing laws that protect the human right of females in the community and use of mass media approaches to create awareness about the existing laws and policies about adolescent sexual and reproductive health related issues may be essential in addressing transactional sexual practices. The policies and laws fall under the societal construct in the ecological model. Transactional sex has been found to be associated with having multiple partners as well as engaging in HIV-related risky behaviour [46]. An earlier study has found high prevalence of HIV infection among people engaged in transactional sex [47]. This high prevalence may not necessarily be due to the high level of exposure as a result of multiple partners [47, 48] but it also creates a situation which makes it impossible for females especially to negotiate for the use of condom as found in this study. Therefore people engaged in HIV prevention must be concern about transactional sex.

### Strategies against unplanned pregnancies, and abortions

From this study, it emerged that respondents believe that some local preparations and herbs are effective abortifacients. Such believes were widespread and well-known thereby resulting in low patronage for modern contraceptives. A study in southern Ghana reported similar believes where there was the widespread notion that ingestion of panacin and cafalgen (painkillers) before sex had some contraceptive effects [49]. Washing of the vagina and vulva with soap and water which is another local practice to avoid pregnancy after unprotected sex has implications on the reproductive health of adolescents. This practice can predispose adolescents to reproductive tract infections which can negatively affect their reproductive functions. Washing the vagina with soap is capable of destroying the normal flora of the vagina and vulva predisposing the female to vaginosis [50]. Vagina cleaning using soap and water has also been reported to increase HIV infection [50, 51]. Health education to community should highlight the negative effects of these practices on the future reproductive health of adolescents.

It is however obvious in the study that these preparations believed to be abortifacients were ineffective as participants in this study indicated that the incidence of unplanned pregnancies was high even among people who had used these items to prevent pregnancy. The findings of this study further show that adolescents who become pregnant do not seek for safe abortion services but engage in unsafe abortion practices using grinded bottles, inserting herbs into the vagina and use of drinks that contain alcohol. These unsafe abortion practices have very serious implications on the health of adolescents as it can result in complications and death. Unsafe abortion is one of the leading preventable causes of maternal mortality across the world [52–55]. Increasing access to safe abortion and comprehensive abortion care were introduced to ameliorate the negative effects of unsafe abortion. Comprehensive abortion care have been found to have high impact in reducing maternal mortality [56–58]. Though Ghana has been implementing comprehensive abortion care in health facilities across all regions [59], the finding of this study reveals a lack of knowledge and awareness about comprehensive abortion care among adolescents as many still engaged in unsafe practices with detrimental effects on their health. More community sensitization should be done to create awareness on the existence of comprehensive abortion care service in health facilities in Ghana.

### Access to reproductive and sexual health services in the community

The study generally found that reproductive health services were available in the community. Also, efforts are being made to bring service close to the communities through outreach programme. However, these efforts were undermined by service-related barriers. Key amongst these was the attitude of health workers towards providing services to adolescents. The study reported there was widespread feeling of negative attitude of service providers towards adolescents, hence their refusal to patronize the services. The negative attitude was reported by both adolescents and stakeholders in this study. This negative attitude was due to community norms and beliefs of health workers concerning some services such as contraceptive use and safe abortion. This will require training of health workers on adolescent-friendly approaches to reproductive health services. Sensitization of community will also be required to increase acceptance. Adolescents’ reproductive health service programmes that target health workers to provide adolescent friendly facility-based services with the approval of community have been found to be more effective [39]. Lack of training has been found to negatively affect the quality of care provided to adolescents in an earlier study [60]. When the attitude of health service providers improves, it will lead to utilization of the services. A study in Kenya found a significant association between friendliness of service provider, and proximity to service provider and uptake of contraceptives [61].

Adolescents in this study were of the view that the designs of reproductive health service outlets did not provide enough privacy. This was therefore a barrier to uptake of such services in the community. Service outlets for adolescent reproductive health services should be designed to provide good privacy. This is because there are socio-cultural norms that prevent adolescent from using reproductive health services. Therefore, adolescents found utilizing reproductive health service risk been described in derogatory terms. This therefore call for measure to ensure strict privacy as that is the only way such services can be patronized by adolescents.

### Limitation of the study

The main limitation of this study is that it was conducted in one rural district in Northern Ghana and the findings cannot be assumed to be the same in other settings. However, the study provides insight into areas to target for health promotion and interventions on adolescent reproductive health choices.

## Conclusions

This study concludes that adolescents in this study generally engaged in risky reproductive health choices with potential of negatively affecting their reproductive health in future. Social and health systems barriers inhibited the utilization of existing reproductive health services. Advocates for reproductive health service providers need to develop better innovative ways to provide this important service to adolescent especially those who are out of school. Sexual and reproductive health promotional activities should target parents as a way of breaking the social barriers. Community sensitization and training of health workers is required to remove barriers and increase the utilization of reproductive health services.

## Abbreviations

FGD: Focus group discussion
GDHS: Ghana Demographic and Health Survey
HIV: Human Immunodeficiency Virus
ICPD: International Conference on Population and Development
IDI: in-depth interview
NACP: National AIDS/STIs Control Programme
STI: Sexual Transmitted Infections
